# Core Steps of Membrane-Bound Peptidoglycan Biosynthesis: Recent Advances, Insight and Opportunities

**DOI:** 10.3390/antibiotics4040495

**Published:** 2015-11-03

**Authors:** Alvin C. K. Teo, David I. Roper

**Affiliations:** School of Life Sciences, University of Warwick, Gibbet Hill Road, Coventry CV4 7AL, UK; E-Mail: a.c.k.teo@warwick.ac.uk

**Keywords:** peptidoglycan, Lipid I, Lipid II, MraY, MurG, Lipid II flippase, FtsW, MurJ, undecaprenyl pyrophosphate phosphatases

## Abstract

We are entering an era where the efficacy of current antibiotics is declining, due to the development and widespread dispersion of antibiotic resistance mechanisms. These factors highlight the need for novel antimicrobial discovery. A large number of antimicrobial natural products elicit their effect by directly targeting discrete areas of peptidoglycan metabolism. Many such natural products bind directly to the essential cell wall precursor Lipid II and its metabolites, *i.e.*, preventing the utlisation of vital substrates by direct binding rather than inhibiting the metabolising enzymes themselves. Concurrently, there has been an increase in the knowledge surrounding the proteins essential to the metabolism of Lipid II at and across the cytoplasmic membrane. In this review, we draw these elements together and look to future antimicrobial opportunities in this area.

## 1. Introduction

**Figure 1:**
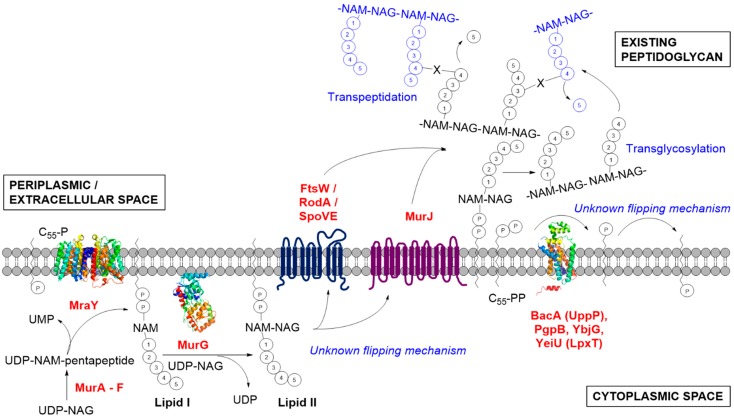
Peptidoglycan biosynthesis in bacteria, highlighting the membrane-associated stage and the several key enzymes discussed in this review. NAM represents *N*-acetylmuramic acid; NAG represents *N*-glucosamine, the repeating disaccharide motif of the peptidoglycan polymer; X represents the canonical 3-4 peptide crosslink between two stem peptides. Enzymes with available crystal structures are shown: MraY (PDB code: 4J72); MurG (PDB code: 1NLM); PgpB (PDB code: 4PX7).

Antimicrobial resistance undermines healthcare on an enormous scale with an associated societal and economic impact that is finally starting to influence policy at a governmental level, prompting the re-engagement of the pharmaceutical industry in this area [1,2,3]. The focus of much attention at the research level has been on the search for novel or reconsidered aspects of bacterial metabolism to target and on the search for drug candidates with the required properties to be a success in the clinic, addressing issues of resistance. There is a growing appreciation that antibiotics that multi-target cellular activities, such as β-lactam inhibition of penicillin binding proteins (PBPs) in the same organism responsible for peptidoglycan biosynthesis, present the most successful opportunities for antibacterial therapeutics [4]. However, β-lactam treatment itself can be subject to resistance through a number of different routes including inactivation of the β-lactam chemical core by corresponding lactamase enzymes or efflux by outer membrane pumps, as seen in many Gram-negative bacterial pathogens. In addition, β-lactam resistance can be achieved by alternative peptidoglycan stem peptide crosslinking chemistry, utilising 3-3 l,d-transpeptidase enzymes (compared to the PBPs above which are generally considered to be responsible for the formation of 3-4 crosslinks between stem peptides, Figure 1) that are unaffected by β-lactam [5,6], the acquisition of β-lactam-insensitive transpeptidase PBPs as seen in methicillin-resistant *Staphylococcus aureus* (MRSA) [7], or target-mediated resistance by gene transfer and recombination in naturally transformable bacterial strains as seen in *Streptococcus pneumoniae* [8,9] and *Neisseria gonorrhoeae* [10].

The enzymes in the bacterial peptidoglycan biosynthetic pathway have been explored as potential drug targets in detailed studies by many research groups and pharmaceutical companies in the past. Some natural products including fosfomycin and d-cycloserine target intracellular proteins in the pathway, but issues of membrane permeability and effective intracellular concentrations have prevented development of high affinity inhibitor lead compounds [11]. However, the fact that peptidoglycan has no counterpart in mammalian cells minimises potential drug toxicity issues and makes it attractive, in theory, for the development of antimicrobial agents [12,13]. Peptidoglycan is a discontinuous, mesh-like, heteropolymeric macromolecule, either located outside the cytoplasmic membrane of Gram-positive bacteria or in-between the inner and outer membrane of Gram-negative bacteria. This fundamental component of the bacterial cell wall is indispensable for preserving cellular integrity by providing structural support, maintaining the cell shape, and protecting the cell from internal turgor pressure. It is essential that new cell walls be formed prior to the division process. If the formation of a functional cell wall is hindered, the cell division process can be effectively interrupted [14].

The biosynthesis of peptidoglycan can be generally divided into three stages (Figure 1). The first stage results in the synthesis of uridine diphosphate-*N*-acetylmuramyl-pentapeptide (UDP-Mur*N*Ac-pentapeptide) in the cytoplasm. This process is initiated in the cytoplasm, wherein UDP-*N*-acetylglucosamine (UDP-Glc*N*Ac) is converted by MurA and MurB to UDP-*N*-acetylmuramic acid (UDP-Mur*N*Ac). The final cytoplasmic peptidoglycan precursor, UDP-Mur*N*Ac-pentapeptide, is generated by the sequential action of adenosine triphosphate (ATP)-dependent ligases, MurC through to MurF [15]. The second stage initiates with the joining of the soluble precursor to a membrane-bound undecaprenyl phosphate (C_55_-P) carrier lipid via the integral membrane protein MraY to form Lipid I on the intracellular face of the cell membrane. Lipid I is then glycosaminylated by a membrane-associated glycosyltransferase, MurG, with UDP-Glc*N*Ac to yield Lipid II [16,17]. In Gram-positive species, Lipid II is modified by the aminoacylation of the ε-amino group of the third amino acid residue or the amidation of glutamate at position 2 to isoglutamine. These modifications have been shown to be essential for subsequent crosslinking [18]. Lipid II is translocated through the cytoplasmic membrane to enable the initiation of the third stage of peptidoglycan biosynthesis. Thereafter, Lipid II is polymerised by glycosyltransferase enzymes, including the essential class A PBPs, prior to peptide crosslinking via the transpeptidase activity of class A and B PBPs, to produce a highly crosslinked peptidoglycan structure [19,20]. It is these latter membrane-bound steps that may offer promise for future drug discoveries in peptidoglycan biosynthesis (Figure 1). The very recent discovery of the Gram-positive specific antibiotic teixobactin with no reported resistance mechanism that binds to Lipid II highlights the important role the lipid-linked metabolites play in the development of new antibacterial drugs [21]. This prompts a re-evaluation of the enzymatic route for Lipid II biosynthesis and its metabolism in light of recent advances in our understanding of the key enzymes involved, which is the subject of this review.

## 2. The Formation of Lipid I by MraY

MraY catalyses the formation of Lipid I by transferring the soluble, cytoplasmic-derived UDP-Mur*N*Ac-pentapeptide onto the C_55_ lipid carrier presented at the cytoplasmic membrane interface [22]. MraY belongs to the superfamily of polyprenyl-phosphate *N*-acetyl hexosamine 1-phosphate transferase (PNPT) [23], including members like WecA, which catalyses the analogous function of transferring *N*-acetylglucosamine 1-phosphate (Glc*N*Ac-1-P) onto the lipid carrier during the synthesis of enterobacterial common antigen and O-antigen lipopolysaccharide in Gram-negative bacteria [24].

In the past, structural and functional studies of MraY have generally proven difficult using conventional overexpression and purification strategies for isolation of this integral membrane protein [22,25]. Whilst it is a multiple-membrane-spanning protein, MraY has both the amino (N-) and carboxyl (C-) termini located in the periplasmic region [26,27]. Attempts to purify quantities of MraY from *Escherichia coli* have met with low levels of overexpression in general, but the sufficient quantities of the *Bacillus subtilis* MraY were purified for study using radioactive-based detection systems [22,25] and Förster resonance energy transfer (FRET)-based assays have been developed for MraY to facilitate drug discovery [28].

In attempts to overexpress and purify MraY in its active form, Ma *et al.* reported the successful implementation of cell-free production of this protein from both *E. coli* and *B. subtilis* origin [29]. Interestingly, functional *E. coli* MraY can only be produced in the presence of membrane lipids, highlighting the importance of membrane lipids to yield a functionally folded and active enzyme. Roos *et al.* subsequently reported the co-translation of *E. coli* MraY with pre-formed nanodiscs in a cell-free expression system and managed to characterise its functional folding and activity [30]. They discovered that the lipid head group chemistry and the degree of lipid saturation used in the nanodisc system will impact the functionality of MraY.

**Figure 2:**
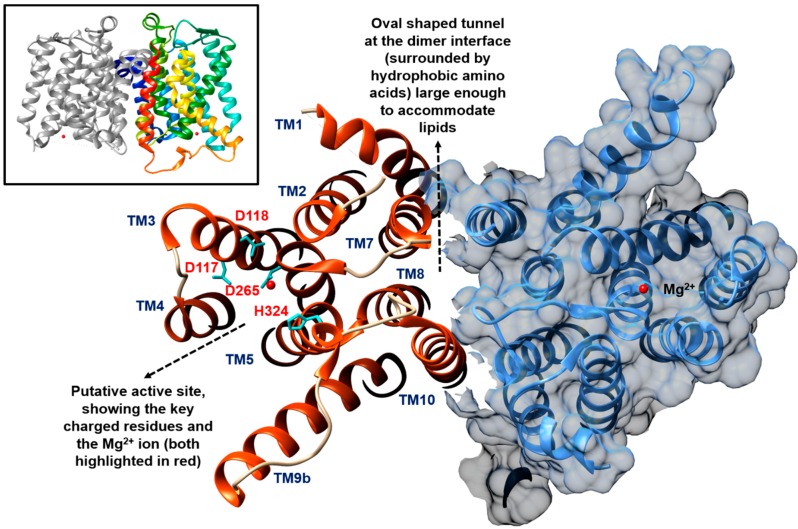
Crystal structure of *A. aeolicus* MraY (PDB code: 4J72) showing a cutaway view from the cytoplasmic side, presenting the key active residues and the oval-shaped hydrophobic tunnel at the dimer interface said to be able to accommodate lipids. The MraY dimer (coloured only for one of the protomers) is shown in the top insert. The figure was prepared using UCSF Chimera version 1.10.1 [32].

Considering the challenges to overexpress and purify this membrane translocase, the first crystal structure of MraY was a landmark event in the field as described at 3.3 Å resolution by Chung *et al.* [31] (PDB code: 4J72, Figure 2). The protein structure was solved from the thermophilic bacterium *Aquifex aeolicus* due to its increased thermal and biochemical stability after screening 19 MraY proteins from different bacteria species. They demonstrated that *A. aeolicus* MraY crystallises as a dimer with a hydrophobic tunnel, postulated to be large enough to accommodate lipids at the centre of the dimer interface. Crosslinking studies conducted in both detergent micelle and lipid membrane conditions substantiated the oligomeric status of *A. aeolicus* MraY.

The crystal structure is consistent with previous topological studies [26], with 10 transmembrane (TM) helices, an interfacial helix, a periplasmic β-hairpin, a periplasmic helix, and five cytoplasmic loops, with both N- and C-termini located in the periplasm. The crystal structure revealed that TM9 splits into two helical fragments (termed TM9a and TM9b), whereby TM9b was deemed to protrude about 20 Å into the lipid membrane (away from the rest of the structure) with a 50° bend relative to the membrane normal [31]. The active site was postulated to be within the cleft established around the inner leaflet membrane region of TM8 when TM5 is surrounded by TM3, TM4, TM8, and TM9b [27,31]. Many of the polar and charged invariant residues identified in *B. subtilis* MraY by Al-Dabbagh *et al.* [27] were located within this cleft by mapping sequence conservation. The catalytic roles of D117, D118, D265, and H324 located in the putative active sites were inferred and supported by mutational studies (Figure 2).

It is known that Mg^2+^ is essential for the activity of MraY [25,27]. Based on similarity to the Mg^2+^ binding motif (DDXXD/N) of farnesyl diphosphate synthases, Lloyd *et al.* suggested that the aspartate pairs, D115 and D116 of *E. coli* MraY are involved in the binding of this divalent cation [25]. However, this notion was challenged by Al-Dabbagh *et al.* for the corresponding pairs, D98 and D99, in *B. subtilis* MraY [27]. Instead, they counter-suggested that D98 is involved in deprotonation of the lipid substrate. The D265 residue of *A. aeolicus* MraY was identified as the coordination site for Mg^2+^ through anomalous scattering studies [31]. An inverted U-shaped groove surrounding TM9b that extends into the active site harbouring D117, as revealed by surface representation of the *A. aeolicus* MraY crystal, was said to be the binding site for the membrane-embedded lipid carrier. The lipid carrier was considered to fit into this groove due to the elastic nature of its polyisoprenyl tail. Being surrounded by conserved charged residues like K121, K133, and D265, together with the Mg^2+^ cation, D177, which corresponds to D98 in *B. subtilis* MraY, was predicted to bind the phosphate moiety of C_55_-P [31]. The fifth cytoplasmic loop of *A. aeolicus* MraY which contains a portion of TM9b (due to its protrusion) connects TM9b and TM10. The conserved sequence specific to the PNPT superfamily, *i.e.*, the HHH motif, was identified in this region, which was speculated to be the binding site for UDP-Mur*N*Ac-pentapeptide [31].

The catalytic mechanism of MraY is yet to be confirmed. A two-step mechanism was first proposed by Heydanek *et al.*, involving the attack of an active site nucleophile upon the β-phosphate of the UDP-Mur*N*Ac-pentapeptide to form a covalent Mur*N*Ac-pentapeptide-phosphoenzyme intermediate, whilst releasing uridine monophosphate (UMP) [33]. It is then followed by the attack by an oxyanion from C_55_-P on the phosphoenzyme intermediate to regenerate the free MraY enzyme with the formation of Lipid I [25,34]. Alternatively, a one-step catalytic mechanism was put forward for MraY to involve a direct nucleophilic attack of the phosphate oxyanion of C_55_-P onto the β-phosphate of UDP-Mur*N*Ac-pentapeptide, resulting in the formation of Lipid I, while releasing UMP in a single step [27].

The availability of a crystal structure can facilitate the studies of the structure-activity relationship of a target protein, serving as an important starting point for synthetic drug development [35,36]. Synthetic protein inhibitors with enhanced potency and efficacy can, in theory, be developed by probing into the atomic structure of the target enzyme, specifically the substrate-binding sites. The cytosolic enzymes involved in peptidoglycan biosynthesis, such as GlmS, GlmM, GlmU, and MurA-MurF that had been crystallised, have been the subjects of intense drug discovery efforts. However, whilst many inhibitors have been described, no major class of clinical candidate or licensed drug has been produced [11]. The key membrane proteins reviewed here may harbour some interesting new targets, but challenges still prevail in terms of biochemical and biophysical studies of these proteins. To advance the knowledge of MraY, the critical membrane translocase that forms Lipid I, co-crystallisation involving its natural substrates (*i.e.*, UDP-Mur*N*Ac-pentapeptide or C_55_-P) and/or other ligands, will reveal more information about the substrate-binding mechanism.

MraY is the target for many classes of nucleoside natural product inhibitors, predominantly synthesised by the *Streptomycetes*, including mureidomycins, pacidamycins, napsamycins, and sansanmycins which belong to the peptidyl nucleosides class, and fatty acyl nucleosides like liposidomycins and caprazamycins. It is also inhibited by muraymycins (lipopeptidyl nucleosides) and nucleoside disaccharides, such as tunicamycins, streptovirudines, and corynetoxins, besides the capuramycins (glycosyl nucleosides). Over the years, these MraY inhibitors have been extensively reviewed [34,37] and thus will not be further discussed in this review. Although mureidomycins and muraymycins were found to be effective in animal models [34], none of these compounds are suitable for clinical use, due to issues of toxicity associated with a variety of eukaryotic membrane-associated glycosyltransferases [38]. A novel caprazamycin derivative, caprazene 4-butylanilide (CPZEN-45), is currently in the preclinical study phase for the treatment of tuberculosis [39] targets WecA in *Mycobacterium tuberculosis* and TagO in *B. subtilis* [40]. Both of these membrane proteins are orthologous and belong to the same PNPT superfamily as per MraY [23]. They are involved in the biosynthesis of mycolylarabinogalactan and wall teichoic acid, respectively.

MraY is also targeted by the lysis protein E from bacteriophage ΦX174 [34]. The interaction of lysis protein E with MraY was found to be mediated by the host peptidyl-prolyl isomerase SlyD [34,41]. The F288L mutation in *E. coli* MraY, which is located on TM9, can confer resistance to the lysis protein E [42]. Interestingly, the substantial protrusion in TM9 revealed in the *A. aeolicus* MraY crystal structure positions the corresponding F288 residue in TM9a, close to the outer face of the cytoplasmic membrane. Together with E287, these two residues were said to interact with lysis protein E which harbours the RWXXW motif, and is also found on many other cationic antimicrobial peptides [31,43].

## 3. The Formation of Lipid II by MurG

**Figure 3:**
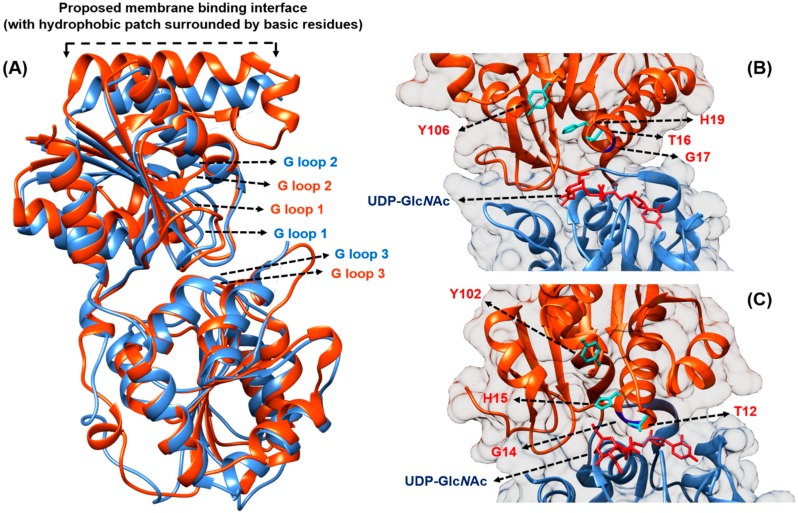
(**A**) Comparison of MurG from *E. coli* (orange, PDB code: 1NLM) and *P. aeruginosa* (blue, PDB code: 3S2U), showing that both structures display high structural similarity despite sharing only 45% sequence similarity. The G-loops (coloured according to bacterial origin) proposed to stabilise negatively charged phosphates from Lipid I and UDP-Glc*N*Ac are shown; (**B**) *E. coli* and (**C**) *P. aeruginosa* MurG in complex with UDP-Glc*N*Ac (red), with key residues being highlighted; the Lipid I-binding N-terminal domain is coloured in orange, while the UDP-Glc*N*Ac-binding C-terminal domain is coloured in blue. The figure was prepared using UCSF Chimera version 1.10.1 [32].

MurG is the membrane-associated glycosyltransferase that catalyses the next lipid-linked step by transferring Glc*N*Ac from UDP-Glc*N*Ac to the C4 hydroxyl of the membrane-anchored Lipid I to form Lipid II [44]. In contrast to MraY, MurG is a soluble protein working at the cytoplasmic membrane surface in order to access its membrane-bound Lipid I substrate. The first MurG crystal structure from *E. coli* was solved by Ha *et al.* at a resolution of 1.9 Å [44] (PDB code: 1F0K). Subsequently, the UDP-Glc*N*Ac-bound crystal of *E. coli* MurG was solved at 2.5 Å by the same group [45] (PDB code: 1NLM, Figure 3). These structures were joined by that of the *Pseudomonas aeruginosa* protein, bound to the sugar substrate, by Brown *et al.* at 2.2 Å resolution (PDB code: 3S2U, Figure 3).

MurG belongs to the glysosyltransferase B superfamily, whose activity is independent of any metal ions [45,46]. MurG utilises a sequential ordered Bi-Bi mechanism whereby the donor substrate, UDP-Glc*N*Ac binds to the enzyme first, before the acceptor Lipid I is recruited to the enzyme for the sugar transfer reaction to occur [45]. Crystal structures revealed that MurG is composed of two domains, linked by a hinge region, separated by a deep cleft of about 20 Å deep and 18 Å across its widest point [44]. The width of the cleft was noted to reduce by 2 Å when the UDP-Glc*N*Ac is bound, with the enzyme adopting a more closed conformation [45].

The C-terminal domain of MurG harbours the binding site for UDP-Glc*N*Ac, while Lipid I binds to the N-terminal domain. MurG is predicted to associate with the inner leaflet of the cytoplasmic membrane through its N-terminal hydrophobic patch, which is surrounded by basic residues, next to the Lipid I binding site (Figure 3). This membrane association is thought to be driven by both hydrophobic and electrostatic interactions with the negatively charged lipid membrane [44]. In the *P. aeruginosa* MurG co-crystal with UDP-Glc*N*Ac, the membrane-association region is disordered. Brown *et al.* thus hypothesised that the interaction with the acceptor Lipid I is probably needed to stabilise MurG in this region [47]. Both the N- and C-domains of MurG present a characteristic Rossmann fold (with α/β open-sheet motif), which is a signature to nucleotide-binding domains, presenting three conserved glycine-rich stretch of amino acids (termed the G-loops), proposed to stabilise the negatively charged phosphates from the substrates, independent of any metal ions [45] (Figure 3). Sequence alignment of MurG homologues pinpointed that the invariant residues are located at or near the cleft between the two domains, thus placing the active site likely along that region [44,47,48].

Despite sharing only 45% overall sequence similarity, MurG from *P. aeruginosa* shares many common features with its *E. coli* counterpart, and their crystal structures were reported to overlap closely [47] (Figure 3). The substrate-binding mode of *P. aeruginosa* MurG is said to resemble that of conformer A in *E. coli* as described by Ha *et al.* [44]. By comparing their crystal structures (PDB codes: 1NLM *vs.* 3S2U), Brown *et al.* noted that the C-terminal domain of the *E. coli* MurG moves away from the N-terminal domain by approximately 15° about the inter-domain hinge, resulting in a significantly wider cleft, displacing the UDP-Glc*N*Ac substrate by about 6 Å [47]. The amide group of G14 in *P. aeruginosa* MurG can form a hydrogen bond to the α-phosphate oxygen of UDP, placing the β-phosphate near to the N-terminal helix 1. This facilitates favourable interaction between the helix dipole with the negatively charged phosphate. In contrast, the equivalent G17 in *E. coli* MurG is positioned closer to the C6-hydroxyl group of the UDP cofactor. This difference is said to stabilise the more closed morphology of the cofactor binding site in *E. coli* MurG [47] (Figure 3).

The critical residues T16, H19, Y106 identified in *E. coli* MurG to bind Lipid I are also present in the *P. aeruginosa* MurG co-crystal complex, namely T12, H15, and Y102 in equivalent (Figure 3). These residues were found to be invariant in other MurG homologues across bacterial species. In particular, the H19 residue located close to the bound UDP-Glc*N*Ac and the proposed Lipid I binding site might be involved in anchoring the polyisoprenyl tail of the C_55_-P acceptor [47]. To propel further structural insight in terms of substrate binding and catalytic mechanism, a co-crystal structure of MurG with the Lipid I substrate is required.

Known inhibitors to the glysosyltransferase enzymes typically possess the nucleoside moiety. Trunkfield *et al.* reported the synthesis of uridine-linked transition state mimics, in which 10 of 18 compounds demonstrated inhibition towards *E. coli* MurG *in vitro* [49]. On the other hand, the phenotypic screening of small synthetic compounds to potentiate the activity of β-lactams to MRSA led to the discovery of a steroid-like compound named murgocil by Mann *et al.* [50]. This compound was found to selectively hinder peptidoglycan biosynthesis in *S. aureus* cells by the inhibition of MurG. In the presence of murgocil, Lipid II synthesis was reduced in a dose-dependent manner and stable murgocil-resistant mutants were found to map to single non-synonymous mutations in MurG. The four identified mutation sites are located near the uracil binding pocket, lining the cleft between the two domains. *In silico* structural modeling using the *E. coli* MurG co-crystal structure as a template demonstrated that murgocil binds to the deep cleft separating the two domains of the enzyme. It was postulated to compete with the binding site of UDP-Glc*N*Ac, which may lock the enzyme into a rigid, non-active form [50].

Murgocil was shown to not affect the membrane association of MurG. It was previously reported that Lipid II mediates the localisation of the class A bifunctional PBP2 in *S. aureus* [51]. Mann *et al.* observed that PBP2 was highly delocalised in murgocil-treated cells and the delocalisation was demonstrated to be murgocil-dependent, not as a result of the general disassembly of the divisome [50]. In essence, murgocil-dependent delocalisation of PBP2 involves the depletion of Lipid II rather than the disruption of the FtsZ ring. The synergistic effect of murgocil towards β-lactams can be explained by the delocalisation of PBP2 that renders the cells more susceptible to reduced level of β-lactams to neutralise the remaining functional PBP2 at the septum. The activity of murgocil is specific but restricted to the Gram-positive *Staphylococci* in this study as murgocil lacks bioactivity towards other bacterial species tested [50].

## 4. The Identity of the Lipid II Flippase

Following the formation of Lipid II, this key lipid-linked peptidoglycan building block is translocated (flipped) across the cytoplasmic membrane for the subsequent reaction by the peptidoglycan synthases. The metabolism of Lipid II must be a rapid and continuous process, as calculations have estimated that there are only a few hundred molecules of Lipid II present in the cell membrane at any time [17]. In addition, we know that the carrier lipid C_55_-P is used in a number of other membrane-specific metabolic processes, setting an imperative for its rapid recycling and utilisation [52]. The identity of the flippase(s) for Lipid II has been a contentious subject in the field for decades, and in the past few years two distinct schools of thought have dominated the field.

### 4.1. Contender I: FtsW 

**Figure 4:**
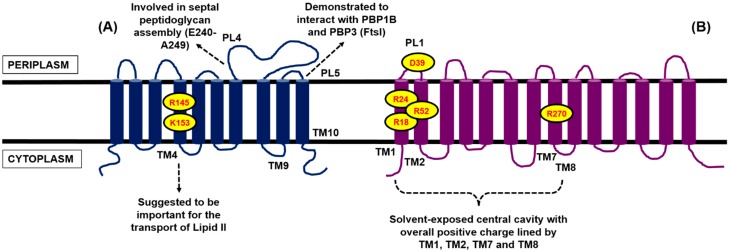
Two-dimensional topology representation for (**A**) FtsW and (**B**) MurJ from *E. coli*, generated based on the topological information presented by Lara and Ayala [54] for FtsW and Butler *et al.* [60] for MurJ, respectively. The several proposed key residues and transmembrane segments important for the putative function of both proteins are highlighted.

In the search for the flippase, Mohammadi *et al.* were the first group to demonstrate biochemically that the translocation of Lipid II involves FtsW by exploiting fluorescence and FRET-based assays [53]. FtsW is an essential polytopic membrane protein in *E. coli* and many other species [54,55,56]. Even though the crystal structure of FtsW is currently unavailable, the topology of this protein from *E. coli* and *S. pneumoniae*, which contain 10 predicted transmembrane helices, has been mapped [54,55] (Figure 4). Importantly, FtsW constitutes a core element of the divisome and had been shown to move to the septum during cell division in *E. coli* and interact with two PBPs, *i.e.*, the class A bifunctional PBP1B that utilises Lipid II directly and the class B transpeptidase PBP3 (also known as FtsI), through the periplasmic loop in-between TM9 and TM10 (Figure 4). This observation was demonstrated by various biochemical techniques including protein-protein interaction assays [57,58,59]. In addition, the gene encoding for FtsW is located on the same division and cell wall (dcw) cluster, in close proximity to MurG and PBP3 [54,55].

A group led by Eefjan Breukink (Utrecht University) had previously established that the translocation of Lipid II is neither a spontaneous flip-flop process utilising its undecaprenyl chain, nor is it induced by the presence of helical transmembrane peptides. The formation of Lipid II is also not coupled to its simultaneous translocation across the cytoplasmic membrane and MurG does not mediate this translocation [61]. Using *E. coli* membrane vesicles, they showed that the transport of Lipid II does not depend on energy sources like ATP or proton motive force, which led to the conclusion that the translocation of Lipid II does not involve any ATP-binding cassette (ABC)-like transporter. However, the flipping of Lipid II across the membrane bilayer is directly linked to the downstream transglycosylation activity whereby the disaccharide-pentapeptide moiety is attached onto existing glycan chains which may drive the reaction by a mass action principle [61].

They went on to demonstrate that the overexpression of both *E. coli* and *S. pneumoniae* FtsW considerably increased Lipid II translocation across the bacterial membranes using fluorescently labelled Lipid II. Purified FtsW proteins reconstituted in the model membrane system were also capable of flipping the derivatised Lipid II [53]. On the contrary, control proteins like MraY and MurJ [62] did not result in the same observed Lipid II translocation phenomenon. When the experiments were repeated with bacterial membranes depleted of FtsW, they observed a reduction (but not abolishment) in Lipid II translocation, supporting the role of FtsW as the putative Lipid II flippase. The remaining transport activity was suggested to be the presence of the FtsW homologue RodA in the membranes [53].

To further elucidate the role of FtsW as the Lipid II flippase, Mohammadi *et al.* discovered that the TM4 of FtsW is required for the translocation, in particular two charged residues, R145 and K153, within this transmembrane segment by mutational studies [63] (Figure 4). The two mutants were not able to complement an *E. coli* FtsW temperature-sensitive strain at the non-permissive temperature (42 °C), hinting at an impairment in cell wall assembly, which is consistent with the postulated function of FtsW as the Lipid II flippase. However, the same mutations did not affect the localisation of FtsW to the division septum [63]. By studying Lipid II analogues differing in sizes and head groups, a size-restricted pore-like structure in FtsW was suggested to accommodate the putative lipid-linked substrate. A particular analogue with a polyethylene glycol (PEG) linker was successfully transported by FtsW, indicating that branched stem peptide Lipid II molecules, including the pentaglycine side chain found in *S. aureus* Lipid II, can be accepted [63].

FtsW was also shown to facilitate the transmembrane movement of different phospholipids, such as phosphatidylethanolamine, phosphatidylglycerol, and phosphatidylcholine, which are common during membrane biogenesis, thus supporting the previous notion that the biosynthesis of peptidoglycan requires ongoing phospholipid synthesis [63,64]. Nonetheless, the two specific charge residues, R145 and K153, in TM4 were not required for this activity and no headgroup specificity was displayed by FtsW in terms of phospholipid translocation [63]. They proposed that given the putative role of FtsW as the Lipid II flippase (during cell division), other members of the shape, elongation, division, and sporulation (SEDS) superfamily such as RodA and SpoVE might also participate in the translocation of Lipid II during cell elongation and sporulation, respectively [64,65]. However, further experimental evidence is needed to substantiate this inference.

### 4.2. Contender II: MurJ 

MurJ (previously designated as MviN) was first identified by Natavidad Ruiz (Ohio State University) through a bioinformatics screen in search of the Lipid II flippase in *E. coli* [62]. MurJ, found to be essential in *E. coli*, belongs to the multidrug/oligosaccharidyl-lipid/polysaccharide (MOP) exporter superfamily, wherein some members within this family had been shown mechanistically as transporters [66]. This discovery was independently supported from experimental findings by Inoue *et al.* around the same time [67]. Subsequently, the MurJ homologue in *Burkholderia cenocepacia*, BCAL2764, was identified by Mohamed and Valvano, and found to be essential for survival, akin to the *E. coli* counterpart [68]. The *E. coli* and *B. cenocepacia* cells depleted of MurJ, in which protein expression was under the control of an inducible promoter [62,68] or controlled by use of a thermosensitive mutant [67], became heterogenous in shape, leading to the formation of ghost cells and eventual lysis. The BCAL2764-depleted strain also became sensitised to β-lactams [68].

The MurJ-depleted cultures only synthesised about one-third of the amount of mature peptidoglycan, with concurrent accumulation of the peptidoglycan lipid and nucleotide precursors [62,67,68]. Utilising a radiolabelled assay, the accumulation of Lipid II intermediates was observed in the thermosensitive MurJ mutant, pointing to its role in the metabolism of the lipid intermediates of peptidoglycan biosynthesis. The growth defect was suppressed with the overexpression of undecaprenyl pyrophosphate (C_55_-PP) synthase (UppS), a *cis*-prenyltransferase that supplies the cells with the C_55_ carrier lipids (in pyrophosphate form) [67]. The two MurJ proteins from *E. coli* and *B. cenocepacia* were demonstrated to be functionally similar, by reciprocal complementation experiment, in restoring the viability in conditional mutants of both species [68].

Like FtsW, the topology for *E. coli* MurJ had been mapped, consisting of 14 transmembrane domains, with both its N- and C-termini in the cytoplasm [60] (Figure 4). In the absence of the crystal structure for MurJ, Butler *et al.* generated a structural model for *E. coli* MurJ based on several crystallised members of the MOP exporter superfamily *in silico* [60]. The structural model of MurJ revealed a solvent-exposed cavity with overall positive charge, lined by TM1, TM2, TM7, and TM8 within the plane of the cytoplasmic membrane. Specific charged residues within this central cavity, *i.e.*, R18 and R24 (TM1), R52 (TM2), R270 (TM8), and D39 (periplasmic loop 1), were demonstrated by mutational studies to be essential for the function of MurJ (Figure 4). This central cavity architecture of MurJ is said to be conserved among the crystallised members of the multidrug and toxic compound extrusion (MATE) transporters family (part of the MOP exporter superfamily), suggesting similar transport function. MurJ was predicted to adopt an outward-opened conformation with two side portals that open into the periplasmic space [60].

An analogous structural model generated for BCAL2764 in *B. cenocepacia* also exhibited the characteristic central cavity with essential charged residues at the same region as reported in *E. coli* MurJ [68]. By abolishing the charges on R18, R24, and R274 on BCAL2764, a non-functional protein was resulted, which is akin to the observation by Butler *et al.* [60,68]. However, BCAL2764 is still functional when the charges were abolished on D39 and R52, in contrast to *E. coli* MurJ. The eight charged residues found in *E. coli* MurJ (within TM1, 2, 8, and periplasmic loop 1) were further studied by Butler *et al.* [69]. In particular, they found that D39 is essential for the biogenesis (possibly for membrane insertion) and function of MurJ. They also explored the conservation of these eight residues on the Gram-positive homologue YtgP in *Streptococcus pyogenes* [70], and found that only three were similarly positioned and vital for function. They hypothesised that some of the charged residues located within the central cavity are driving the interaction with Lipid II, and are possibly involved in some sort of energy coupling during the transport process [69].

Sham *et al.* presented the *in vivo* evidence that MurJ is the Lipid II flippase [71]. By treating the *E. coli* cells with colicin M (which specifically cleaves the disaccharide-pentapeptide moiety from the C_55_ carrier lipid), the level of the soluble colicin M product was reduced upon MurJ inactivation, and in contrast, the accumulation of lipid-linked peptidoglycan precursors was observed. They concluded that the inactivation of MurJ protected Lipid II resulted from the action of colicin M either through the blocking of Lipid II translocation or by interfering with the import or activity of colicin M. By removing the outer membrane of the *E. coli* cells through spheroplasting, and thus providing direct access for colicin M to the flipped Lipid II, the level of soluble colicin M product was not restored, supporting MurJ as the Lipid II flippase [71]. In stark contrast to the *in vitro* findings by Mohammadi *et al.* [53,63], Sham *et al.* observed that, in the *E. coli*-null RodA mutant depleted of FtsW, the translocation of Lipid II was relatively unperturbed, suggesting that these two SEDS proteins are not involved in the transport of Lipid II. However, they observed a reduction in the level of peptidoglycan lipid intermediates in this mutant, implicating that the loss of these SEDS proteins might have an effect on the recycling of C_55_-P or the synthesis of peptidoglycan precursors [71].

MurJ was found to be highly conserved among the Gram-negative *Proteobacteria*, but not for the Gram-positive *Firmicutes*. YtgP, the closest functional homologue to MurJ, is however, conserved in the Gram-positive *Firmicutes*. The *S. pyogenes* YtgP (Spy_0390) was found to complement a MurJ-depleted strain of *E. coli* [70]. Whilst being essential in *S. pneumoniae* and *S. aureus*, YtgP is not essential in the Gram-positive model organism *B. subtilis*, alluding to the presence of other functional homologues. Besides YtgP, the other members in the MOP exporter superfamily, namely SpoVB, YkvU, and YabM, were also identified as putative MurJ homologues. Similarly, they were not found to be essential in *B. subtilis.* Mutant cells lacking these proteins did not display the vegetative defects expected if they serve as the Lipid II flippase like MurJ [72,73].

Fay and Dworkin showed that *E. coli* MurJ was able to complement the sporulation defects in *B. subtilis* cells lacking SpoVB and restored spore production. On the other hand, basal expression of SpoVB and YtgP (in the non-induced state) complemented the growth defect in a MurJ-depleted strain of *E. coli*, but not when the two genes were fully induced [72]. The observation that these two MurJ homologues were not essential in *B. subtilis* led to the speculation that they might be playing an accessory role or are involved in an alternate pathway of peptidoglycan biosynthesis, *i.e.*, flipping lipid-linked precursors, but not necessarily with Lipid II. They could also be involved in the process of cell wall modification, in which the C_55_ lipid carrier is utilised [72,73]. This offered a possible explanation regarding the lethality being observed when YtgP and SpoVB were fully induced in *E. coli* cells depleted of MurJ, due to the sequestration of the C_55_ isoprenoid chain [72].

Meeske *et al.* further investigated the knockout effect of all 10 MOP exporter superfamily members in *B. subtilis*, including YtgP, SpoVB, YkvU, and YabM [74]. The existence of an alternate flippase was alluded to when the decuple mutant was shown to be viable with a similar growth rate as the wild types, with only minimal morphological defects. An uncharacterised gene, *ydaH* (being renamed to *amj*, representing an “alternate to MurJ”), was discovered thereafter to be the synthetic lethal pair to YtgP in *B. subtilis*. When both Amj and YtgP were depleted in *B. subtilis*, aberrant cell morphologies and extensive cell lysis were observed, analogous to the phenotype of MurG knockdown. Amj was also demonstrated to complement *E. coli* cells lacking MurJ. In addition, the expression of either *B. subtilis* YtgP or Amj in *E. coli* cells can support the transport of Lipid II across the cell membrane when the native MurJ is inactivated, using the same colicin M flippase assay as per Sham *et al.* [71,74]. Being only half the size of typical MOP exporter superfamily members, Amj (predicted to contain only six transmembrane segments) is hypothesised to dimerise to form the channel for Lipid II transport in line with the charged central cavity reported in *E. coli* MurJ [60,74].

Last but not least, SAV1754, the MurJ homologue in *S. aureus*, was identified by Huber *et al.* via a chemical genetic approach utilising high throughput screening of a synthetic chemical library for compounds to restore the activity of carbapenem class β-lactams against MRSA [75]. Two structurally related indole compounds, 3-{1-[(2,3-Dimethylphenyl)methyl]piperidin-4-yl}-1-methyl-2-pyridin-4-yl-1*H*-indole (DMPI) and 2-(2-Chlorophenyl)-3-[1-(2,3-dimethylbenzyl) piperidin-4-yl]-5-fluoro-1*H*-indole (CDFI), were discovered to selectively target SAV7154. Fluorescent staining of nascent peptidoglycan revealed that *S. aureus* cells treated with both compounds displayed a significant reduction in overall fluorescence of new cell wall material and a low level of cells showed clear septum staining. The genetic inactivation of SAV1754 resulted in pronounced hypersensitivity towards β-lactams, exceeding the effect of PBP2 inactivation. They asserted that SAV1754 is not likely to play a role in drug import or efflux because the three resistant mutants only conferred marked resistance to the above-mentioned indole compounds without cross-resistance to other cell wall inhibitors tested such as vancomycin, fosfomycin, and moenomycin [75]. Key features of the two different proposed Lipid II flippase proteins are summarised in Table 1.

The identity of the Lipid II flippase(s) remains hitherto a “Holy Grail” in bacterial cell wall biology to complete a core understanding of the lipid-linked stage of peptidoglycan biosynthesis. Given the high metabolic requirement for Lipid II and its carrier lipid, the possibility that the transport of Lipid II involves more than one protein should also be given fair consideration. Structural elucidation of the two contending protein families will provide a vital part of this investigation and may offer more insight into the translocation mechanism involved and possibly the energy source used in this remarkable process.

**Table 1:** Comparison of the two major Lipid II flippase contenders.

Protein	Key Features
FtsW	Essential and conserved among cell wall synthesising eubacteria such as *E. coli* [54,55,56]
	Topology mapped for *E. coli* and *S. pneumoniae*, with 10 TM helices, both N- and C-termini in the cytoplasm [54,55]
	Demonstrated to interact with PBP3 (FtsI) and PBP1B through periplasmic loop between TM9 and TM10 [57,58,59]
	Demonstrated to flip fluorescently labelled Lipid II *in vitro* using bacterial and model membranes [53], R145 and K153 on TM4 are implicated in the translocation of Lipid II [63]
	Demonstrated to flip phospholipids and can accommodate modified Lipid II with a PEG linker [63]
	SEDS superfamily homologues RodA and SpoVE suggested for similar translocation function in cell elongation and sporulation respectively [64,65]
MurJ	Broadly conserved in eubacteria and essential for bacteria such as *E. coli* and *B. cenocepacia* [62,67,68]
	Topology mapped for *E. coli* MurJ with 14 TM helices, both N- and C-termini in the cytoplasm [60]
	Structural model generated *in silico* for *E. coli* and *B. cenocepacia* MurJ, harbouring a central hydrophilic cavity with charged resides such as R18, R24, and R270/R274 implicated for substrate binding and transport function [60,68,69]
	Demonstrated to flip radiolabelled Lipid II *in vivo* [71]
	Functional MOP exporter superfamily homologues like YtgP and SpoVB are not essential in *B. subtilis* [72,74]
	Alternative flippase: Amj discovered to form synthetic lethal pair to YtgP in *B. subtilis* and demonstrated to flip Lipid II [74]

## 5. The Metabolism of C_55_-P: Multiple Enzymes Can Dephosphorylate C_55_-PP

Undecaprenyl pyrophosphate is formed *de novo* on the cytoplasmic side of the cell membrane via the successive addition of eight isopentyl pyrophosphates (in *cis* configuration) onto a molecule of farnesyl pyrophosphate (in *trans* configuration) catalysed by the cytosolic enzyme UppS [76]. The resulting C_55_-PP would have to be dephosphorylated into the monophosphate form to be used as the active carrier molecule in the biosynthesis of peptidoglycan and other cell wall polysaccharides, including teichoic acids, lipopolysaccharides, and enterobacterial common antigens [6,16,52]. Thus, the metabolism of this limited yet common glycan lipid shuttle remains a crucial control point to prevent any imbalance in the formation of the bacterial cell envelope. The current knowledge, mechanism, and metabolism surrounding the C_55_ carrier lipid were recently reviewed in depth by Manat *et al.* [52]. 

UppP (previously known as BacA) was demonstrated by El Ghachi *et al.* in their biochemical studies to be the primary phosphatase that acts on C_55_-PP in *E. coli*, accounting for 75% of total cellular C_55_-PP dephosphorylation [77]. *E. coli* UppP requires divalent metal ions such as Mg^2+^ or Ca^2+^ for activity, in order to coordinate the pyrophosphate moiety of C_55_-PP and facilitate nucleophilic attack by making the pyrophosphate a better leaving group [77,78]. They proposed that UppP takes part in the *de novo* biosynthetic pathway to generate C_55_-P by dephosphorylating C_55_-PP formed by UppS, thus suggesting that it acts on the cytosolic face of the cell membrane. This supposition is fueled by the prediction of a large cytosolic loop that is conserved among UppP homologues, based on a seven TM helices model, coupled with the cytosolic location of UppS which supplies the C_55_-PP substrate [79,80]. In addition, the formation of Lipid I was evident when UppP was coupled to MraY [77].

**Figure 5:**
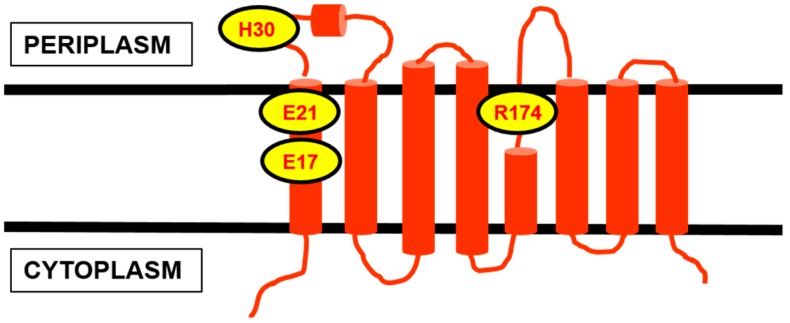
Two-dimensional topology representation for UppP/BacA from *E. coli*, generated based on the topological information of the eight TM helices model presented by Chang *et al.* [78]. The several proposed key residues involved in the binding and catalysis of C_55_-PP are highlighted. The active site of this enzyme is proposed to face the periplasm.

However, Chang *et al.* [78] recently questioned this view by presenting evidence that UppP acts at the periplasmic side of the cell membrane. They demonstrated that the active site of *E. coli* UppP actually faces the periplasm based on their *in silico* structural model, validating it with a molecular dynamics simulation and mutational studies. The molecular modelling data suggested that the pyrophosphate moiety of C_55_-PP sits in the active site pocket surrounded by E17, E21, and R174 (Figure 5), whereas part of its C_55_ chain is situated in a hydrophobic surface in TM2 which facilitates the rearrangement of the long isoprenyl chain of C_55_-PP. The remaining hydrocarbon tail is highly flexible and oriented towards the phospholipid bilayer, akin to the observation of MraY with an inverted U-shaped hydrophobic groove predicted to house the lipid-linked substrate [31,78]. They went on to propose a catalytic mechanism for UppP involving H30 to initiate the nucleophilic attack on the C_55_-PP substrate; E17 or E21, on the other hand, may interact with the pyrophosphate moiety of C_55_-PP through the Mg^2+^ ion. In the putative phosphate binding loop (with the PGXSRSXXT motif), R174 can establish a hydrogen bond with the hydroxyl group of the pyrophosphate moiety [78] (Figure 5).

The overexpression of *E. coli* UppP was reported to result in bacitracin resistance by rapidly exhausting the cellular pool of C_55_-PP. Bacitracin, which is a group of cyclic polypeptide antibiotics, works by sequestering C_55_-PP, thereby preventing its dephosphorylation to occur [77,81,82]. The UppP homologue in *Enterococcus faecalis*, EF2439, was identified by Shaaly *et al.* and found to confer low level bacitracin to *E. faecalis* [83]. Similar to the *E. coli* UppP mutant, the EF2439 mutant displayed no observable growth phenotype in terms of growth rate, colony morphology, and biofilm formation in *E. faecalis*. However, reduced bacitracin tolerance was detected with the mutant [83]. Interestingly, Kjos *et al.* identified UppP as the target for lactococcin G, a class IIb two-peptide bacteriocins targeting *Lactococcus lactis* [84]. Exploiting whole genome sequencing, all 12 lactococcin G-resistant mutants were found to exhibit mutations in or near the gene encoding for UppP. These mutants were also resistant to the related bacteriocin, *i.e.*, enterocin 1071. This similar degree of resistance observed suggests that UppP is the target of both lactococcin G and enterocin 1071. In addition, the expression of *L. lactis* UppP in *S. pnenumoniae* cells, which are normally insensitive to lactococcin G and enterocin 1071, had sensitised the cells to these two bacteriocins [84].

Given the fact that the dephosphorylation activity of UppP does not account to 100% and the *bacA* gene is not essential in *E. coli*, it signifies the presence of other phosphatases that functionally complement UppP [79,82,85]. Upon the translocation of Lipid II across the cell membrane, the C_55_ lipid carrier (now in the pyrophosphate form) has to be dephosphorylated before being recycled back to the cytoplasmic side of the cell membrane to be reused [79,86]. One can thus envisage the need for C_55_-PP phosphatase activity at the periplasmic side to facilitate the recycling of this important but limited shuttle molecule. El Ghachi *et al.* identified three other integral membrane proteins, namely PgpB, YbjG, and YeiU, in *E. coli* that displayed C_55_-PP phosphatase activity and whose overexpression also contributed to bacitracin resistance. The genes encoding these three enzymes can be individually disrupted without any apparent growth defect *in vivo*, but the triple deletion of the *bacA*, *pgpB*, and *ybjG*, genes posed a lethal effect on *E. coli* [82].

Surprisingly, the overexpression of YeiU was not able to rescue the cell viability of the thermosensitive conditional triple mutant (with UppP expression restricted at 42 °C), despite the fact that its overexpression had resulted in a significant dephosphorylation of C_55_-PP [82]. Subsequently, the phosphotransferase function of YeiU (being then renamed to LpxT) was discovered by Touzé *et al.* to transfer the β-phosphate from C_55_-PP to Lipid A, forming Lipid A 1-diphosphate, thus connecting C_55_-P metabolism to Lipid A remodeling [87]. Similarly, PbrB was biochemically characterised to desphosphorylate C_55_-PP in *Cupriavidus metallidurans* with additional phosphotransferase function [88]. It transfers the phosphate group from C_55_-PP to the extruded lead ions (by the action of PbrA, an efflux pump for divalent metal ions), which results in lead precipitation, thus conferring lead resistance to the bacteria.

PgpB, YbjG, and LpxT belong to the type 2 phosphatidic acid phosphatase (PAP2) superfamily, which is distinct to UppP [79,85]. Another member of the PAP2 superfamily is BrcC (previously known as YwoA) from *B. subtilis*, whose overexpression confers bacitracin resistance, with significant elevation of C_55_-PP phosphatase activity [81]. These PAP2 members displayed the consensus motif of the acid phosphatase domain, with signature residues facing the periplasmic region [79]. The three distinct motifs were designated as C1: “KX_6_RP”; C2: “PSGH”; and C3: “SRX_5_HX_3_D” [89].

Among these integral membrane PAP2 members, PgpB is considered the most characterised. Initially discovered for its phosphatase activity towards phosphatidylglycerolphosphate, phosphatidic acid, and lysophosphatidic acid [90,91,92], PgpB was subsequently found to act on diacylglycerol pyrophosphate [93] and undecaprenyl pyrophosphate [82]. The biochemical studies conducted on PgpB from *E. coli* demonstrated a broad substrate spectrum, and diacylglycerol pyrophosphate was considered the preferred substrate for PgpB *in vitro* and was able to stimulate the activity of PgpB on C_55_-PP in a dose-dependent manner [85]. Similarly, other common phospholipids in *E. coli*, such as phosphatidylglycerol, phosphatidylethanolamine, and cardiolipin, contributed to the same stimulation effect *in vitro*. Touzé *et al.* reasoned that the C_55_-PP substrate might have adopted a non-native conformation or orientation in the detergent micelles containing PgpB, thus preventing the charged head group to be presented to the active site of the enzyme. The input of phospholipids in this case could have rendered the detergent micelles more native membrane-like, and thus stimulated the activity of PgpB towards C_55_-PP [85]. The substrate promiscuity of PgpB could serve as a link between C_55_-P and phospholipid metabolism.

**Figure 6:**
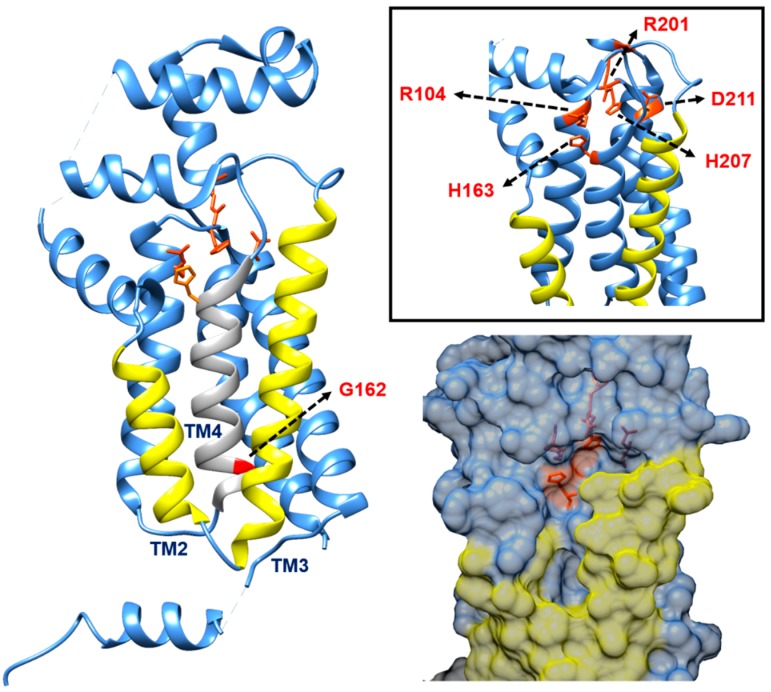
Crystal structure of *E. coli* PgpB (PDB code: 4PX7) showing the proposed V-shaped cleft formed by TM2 and TM3 (highlighted in yellow) for the membrane-associated lipids to enter the enzyme active site facing the periplasmic region. The key catalytic residues discussed are presented here. The figure was prepared using UCSF Chimera version 1.10.1 [32].

With the crystal structure of *E. coli* PgpB being solved recently at 3.2 Å by Fan *et al.*, structural elucidation of this enzyme was facilitated [94] (PDB code: 4PX7, Figure 6). A double point mutant which showed identical properties to the wild-type enzyme was used for crystallisation. *E. coli* PgpB was found to display a similar folding topology and nearly identical active site to the other soluble PAP2 enzymes, but it differs in terms of its substrate-binding mechanism. The binding of lipid substrates is deemed to proceed with an induced fit mechanism. The potential substrate entrance is the cleft formed by a V-shaped transmembrane helix pair, TM2 and TM3, allowing lateral movement of the lipid substrate to enter the active site from the phospholipid bilayer (Figure 6). The catalytic site of this enzyme was proposed to be near the solvent-membrane interface facing the periplasm, with the periplasmic opening side being over 10 Å wide [94].

With respect to the catalytic mechanism for PgpB (and in reference to other PAP2 enzymes), it was proposed that the nucleophilic attack of the β-phosphate of C_55_-PP would be performed by a conserved histidine residue in motif 3 (Figure 6). This produces a phosphoenzyme intermediate that is stabilised by neighbouring arginine residues from the same motif and several other residues in motif 1 and 2 to hold the phosphate group close to the catalytic histidine in motif 3 [79]. Fan *et al.* suggested H207 and D211 in *E. coli* PgpB to form a charge-relay pair for the nucleophilic attack and formation of the phosphoenzyme intermediate, which is stabilised by the H163 that catalyses the cleavage of the phosphate group [94] (Figure 6). The lipid substrate is said to position its phosphate head into the bottom of the active pocket and towards the nucleophilic H207 to form the phosphoenzyme complex, allowing H163 to complete the second step of dephosphorylation by recruiting a water molecule from the solvent-accessible side of the enzyme active site. R104 is said to interact with H207 and potentially with the substrate, while R201 is deemed to interact with H163 (Figure 6). Last but not least, the N-terminal of TM4 is potentially involved in the binding of the phosphate group in a charge-dipole interaction as the lack of side chain of G162 at the N-terminal of this transmembrane helix favours the binding of the phosphate group [94] (Figure 6). Key features of UppP in comparison to several PAP2 members are summarised in Table 2.

**Table 2:** Comparison of UppP/BacA with the three members in PAP2 superfamily.

Protein		Key Features
UppP/BacA		Suggested to be the primary C_55_-PP phosphatase in *E. coli* (75% of total cellular activity) [77]
		Topology and structure modelled *in silico* with eight TM helices, both N- and C-termini in the cytoplasm [78]
		Active site predicted to face the periplasm, with key residues E17, E21, H30, and R174 based on *in silico* modelling [78]
		Dependent on metal ions for activity [77,78]
		Target for bacteriocins [84] but conferred bacitracin resistance when overexpressed [77,83]
PAP2 superfamily	PgpB	Demonstrated activity toward phosphatidylglycerolphosphate, phosphatidic acid, lysophosphatidic acid [90,91,92], diacylglycerol pyrophosphate [93], and C_55_-PP [82]
		Topology mapped [85] and crystal structure from *E. coli* solved [94]
		Active site faces the periplasm, with a V-shaped TM2 and TM3 pair suggested to accept membrane-associated lipid substrates, catalytic triad: H163, H207, and D211 [85,94]
		Not dependent on metal ions for activity [85,92] with broad substrate specificity, capable of utilising a range of lipid pyrophosphates *in vitro* [85]
		Activity towards C_55_-PP *in vitro* (in detergent micelles) stimulated in the presence of phospholipids and diacylglycerol pyrophosphate [85]
	YbjG	Demonstrated activity towards C_55_-PP [82]
		Topology mapped with five TM helices, N-terminus in the periplasm and C-terminus in the cytoplasm [79]
		Active site suggested to face the periplasm [79]
	YeiU/LpxT	Demonstrated activity towards C_55_-PP [82]
		Topology mapped with six TM helices, both N- and C-termini in the cytoplasm [79]
		Active site suggested to face the periplasm [79]
		Demonstrated phosphotransferase function towards Lipid A [87]

**Note:** Triple deletion of *bacA*, *pgpB*, and *ybjG* genes is lethal to *E. coli* [82].

It is important to note that the presence of either one of UppP, PgpB, or YbjG can sustain normal growth in *E. coli* [82], indicating that each of these three membrane phosphatases can presumably participate in both the *de novo* synthesis of C_55_-P and the subsequent recycling of C_55_-PP. Since the above-mentioned enzymes have their active sites predicted to face the periplasm [78,79,85,94], it is therefore not surprising to observe that they can functionally complement each other. However, if C_55_-PP is dephosphorylated on the periplasmic side of the cytoplasmic membrane following the action of class A PBPs and monofunctional glycosyltransferase, then there must be an obligatory flipping of the monophosphate form of the active lipid carrier prior to the recruitment by MraY to form Lipid I. This hypothesis should hold true unless there is another C_55_-PP phosphatase (unknown and yet to be characterised) that can perform such a function at the cytoplasmic side of the cell membrane. It has been postulated that a driving force for the unidirectional transbilayer movement of lipids (by passive diffusion or dynamic rearrangement in the cell membrane) with the accumulation of C_55_-PP on one side of the lipid bilayer would favour its translocation towards the other side, and *vice versa* for the case of C_55_-P [52]. Nevertheless, the presence of a dedicated protein to transport these lipid molecules is a source of uncertainty, particularly given the high metabolic turnover of C_55_-P that is required for a variety of cellular activities.

## 6. Conclusions

Although the pathway for peptidoglycan biosynthesis has been studied for many decades, there is still a great deal to discover concerning the structure and function of many of the enzymes involved and the coordination of their activities. Central to the overall biosynthetic process is the metabolism of Lipid II at and through the cytoplasmic membrane. The nature and energetics of the Lipid II translocation process are still a subject of controversy [95], and despite recent advances in the field, there is still much to decipher. Related to this metabolism is an almost complete lack of understanding of the overall recycling process for undecaprenyl phosphate which is present in limiting amounts in bacterial membranes, yet is used in a variety of different processes in bacteria [52]. There is an interesting analogous situation in eukaryotic membranes whereby dolichol is used as a carrier lipid for the glycan precursor in the early stages of *N*-linked protein glycosylation [96]. The molecular basis of several steps involved in the synthesis and the recycling of dolichol and its derivatives is still unknown, just like the prokaryotic counterpart.

Clearly there is a large number of naturally occurring secondary metabolites/antimicrobials including the recently discovered and heralded teixobactin [21], and whilst these compounds generally target different aspects of the Lipid II structure, it is interesting to speculate whether direct sequestration of Lipid II or a consequent derailment of membrane-associated undecaprenyl phosphate metabolism is actually responsible for the subsequent bactericidal events. Thus, an in-depth understanding of this crucial process will certainly reveal the long-sought-after biological understanding of the process and may also lead to novel antibacterial opportunities targeting this crucial aspect of bacterial metabolism which could be considered an Achilles’ heel.
